# Trismus Following Inferior Alveolar Nerve Block: A Case Report

**DOI:** 10.7759/cureus.73394

**Published:** 2024-11-10

**Authors:** Banine Khalifeh, Karin Simonian, Moustafa Houmani, Abbass El-Outa, Wael Khalil

**Affiliations:** 1 Oral and Maxillofacial Surgery, Faculty of Dental Medicine, Lebanese University, Beirut, LBN; 2 Prosthodontics, Faculty of Dental Medicine, Lebanese University, Beirut, LBN; 3 Dentistry, Observe Clinical Research Group, Beirut, LBN

**Keywords:** inferior alveolar nerve block, limited mouth opening, masticatory muscles, regional anesthesia, trismus

## Abstract

Trismus, characterized by a restricted mouth opening due to involuntary muscle spasms, poses significant challenges to eating, speaking, and other oral functions. In fact, this condition often results from various factors including post-third molar surgery complications, temporalis and medial pterygoid muscle involvement, repeated or incorrectly administered intramuscular injections, and complications from local anesthesia usage. Despite the high safety and efficacy of local anesthetics in oral surgery, side effects including trismus warrant careful consideration. Management strategies for muscle-related trismus typically involve a multitude of early interventions, with resolution generally occurring within few weeks. Our paper presents a case where trismus following inferior alveolar nerve block administration was successfully managed through a comprehensive approach, leading to significant improvements in mouth opening, pain, and edema. Despite being challenging, this case highlights the effectiveness of a multifaceted treatment strategy in managing trismus and underscores the importance of early and appropriate interventions to enhance patient outcomes.

## Introduction

Trismus is a painful condition in which normal mouth opening is restricted due to involuntary muscle contraction or spasm associated with mastication. This can affect eating, speaking and other oral functions. For females and males, the lower limit of normal maximum opening is 35 mm and 40 mm, respectively [1,2].

Trismus can be brought about by several factors, one of which is being a minor complication following third molar surgery; since such a surgery often involves incising along the the ascending ramus, temporalis muscle fibers may be severed, in addition to the medial pterygoid muscle, which is located posterior to the pterygomandibular raphe [2]. Moreover, other factors include intramuscular injections or muscle trauma causing hematoma formation and fibrosis, repeated muscle puncture through injections in the same area within a short period of time (e.g. repetitive inferior alveolar nerve block), or even incorrectly administered inferior alveolar nerve block (IANB) or posterior maxillary injections. Additionally, low-grade infections, needle fractures in the concerned muscles, and large quantities of local anesthetic solution injected into a restricted region resulting in tissue expansion, may contribute to trismus [2,3]. Furthermore, another factor for developing trismus may be the local anesthetic itself; in fact, despite the fact that local anesthetics use in oral surgery is very safe and effective, side effects may still arise and should be taken into consideration, one of which is trismus [4,5].

Several modalities have been suggested to manage muscle-related trismus; soft diet, heat therapy, prescription of antibiotics, anti-inflammatory drugs, analgesics, muscle relaxants, or physiotherapy are methods of early treatment for trismus. Trismus typically resolves in six weeks, with a range of four to 20 weeks [3].

## Case presentation

**Figure 1:**
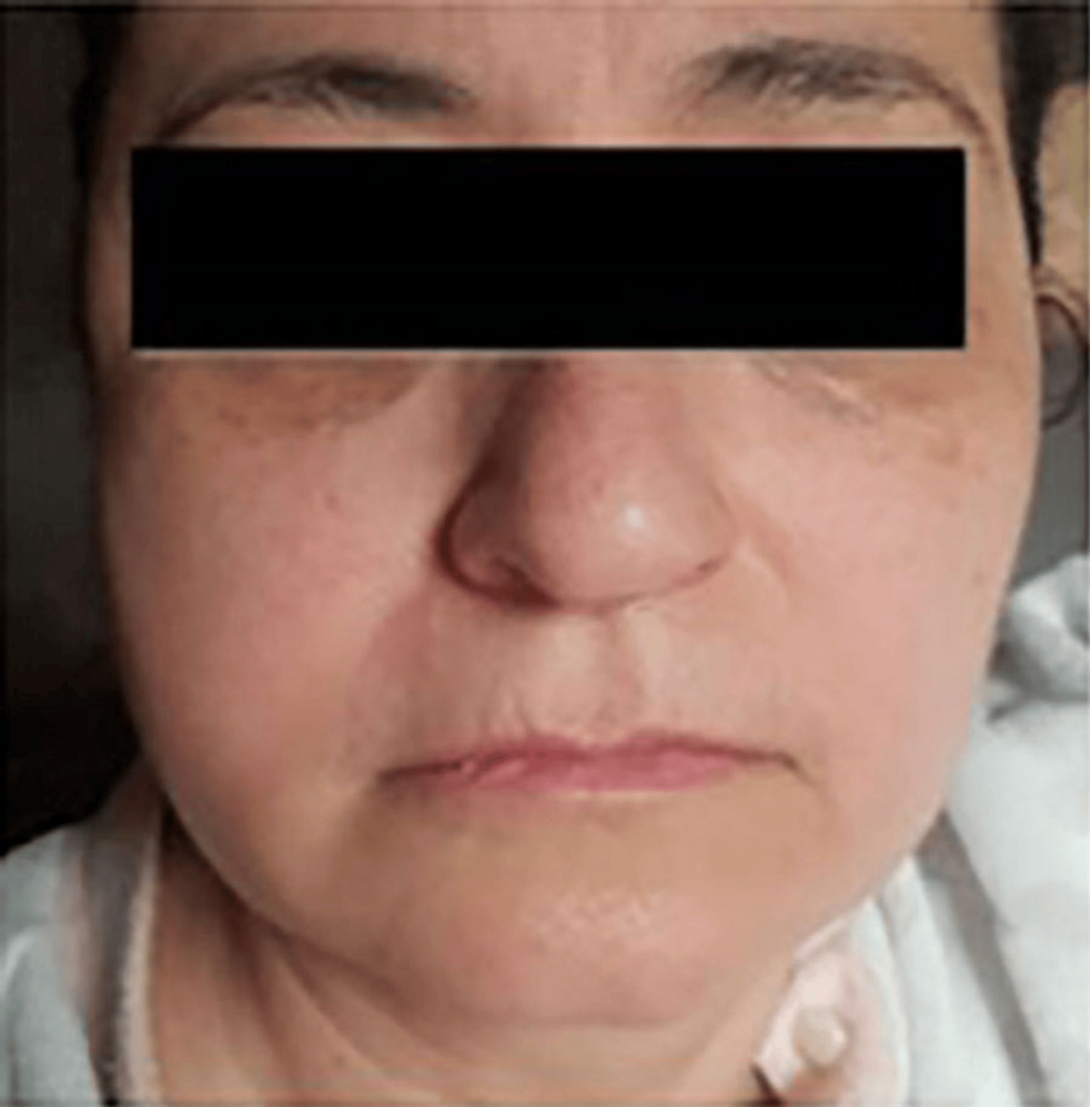
Edema of the right side of the face

**Figure 2:**
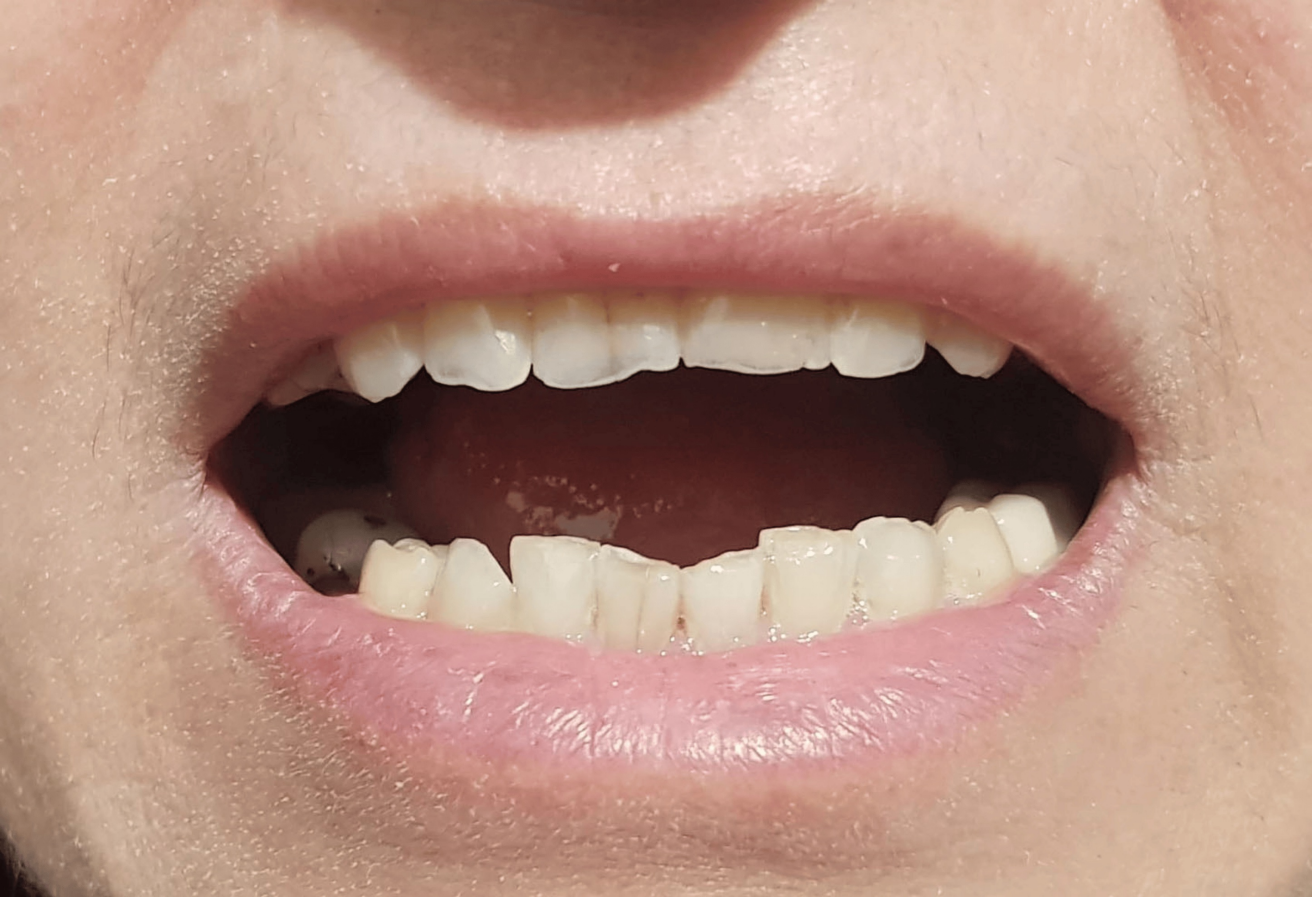
Maximum mouth opening of 18 mm

**Figure 3:**
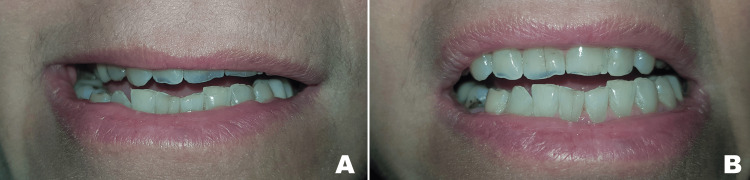
(A) Right lateral movement at 9 mm; (B) left lateral movement at 3 mm

A 59-year-old female with no significant medical history visited the Department of Oral and Maxillofacial Surgery at the Lebanese University, Faculty of Dental Medicine, complaining of new-onset edema on the lower right side of the face with limited mouth opening and pain upon attempting to open more. Upon history taking, patient reported visiting her dentist 12 hours earlier for a routine dental check-up and had undergone a restorative procedure to treat a carious lesion on tooth 47. An inferior alveolar nerve block was performed twice (around 3.4cc) using Persocaine® (2% lidocaine with 1:100000 epinephrine) anesthetic; several hours later, the presenting symptoms started to appear. On examination, right lower face edema was evident (Figure 1). Furthermore, palpation returned moderate pain at the level of the right temporo-mandibular joint and medial pterygoid muscle regions. Concerning her mouth opening, the maximum opening was measured at 18 mm during the first consultation, with restriction in lateral movement measuring 9 mm of right lateral movement and 3 mm of left lateral movement (Figures 2, 3).

After reviewing the results from the clinical exam and the complementary exams, and ruling out possible overlapping conditions, the patient was diagnosed with trismus following IANB. Management initially consisted of warm application every day, with daily exercises along with massaging of the temporomandibular joint (TMJ) area and muscles (Table 1).

**Table 1:** Management protocol followed initially

Exercise	Medication
Mouth opening and closing exercises 10 times/day	Muscerol 2^®^ (paracetamol + orphenadrine): 2 tablets 3 times daily for 7 days
Mouth opening as wide as comfortable and hold the position for 5 seconds, 3 times/day	Profinal^®^ (ibuprofen) 400mg: 1 tablet 3 times daily for 7 day
Lateral right and left movements of the jaw 10 times/day	Reparil^®^ (aescin) 20 mg: 2 tablets 3 times daily for 7 days
Holding of the laterality movement for 5 seconds on each side 3 times/day	
Protrusion movement of the jaw 5 times/day	
Holding of the protrusive position for 5 seconds	

**Figure 4:**
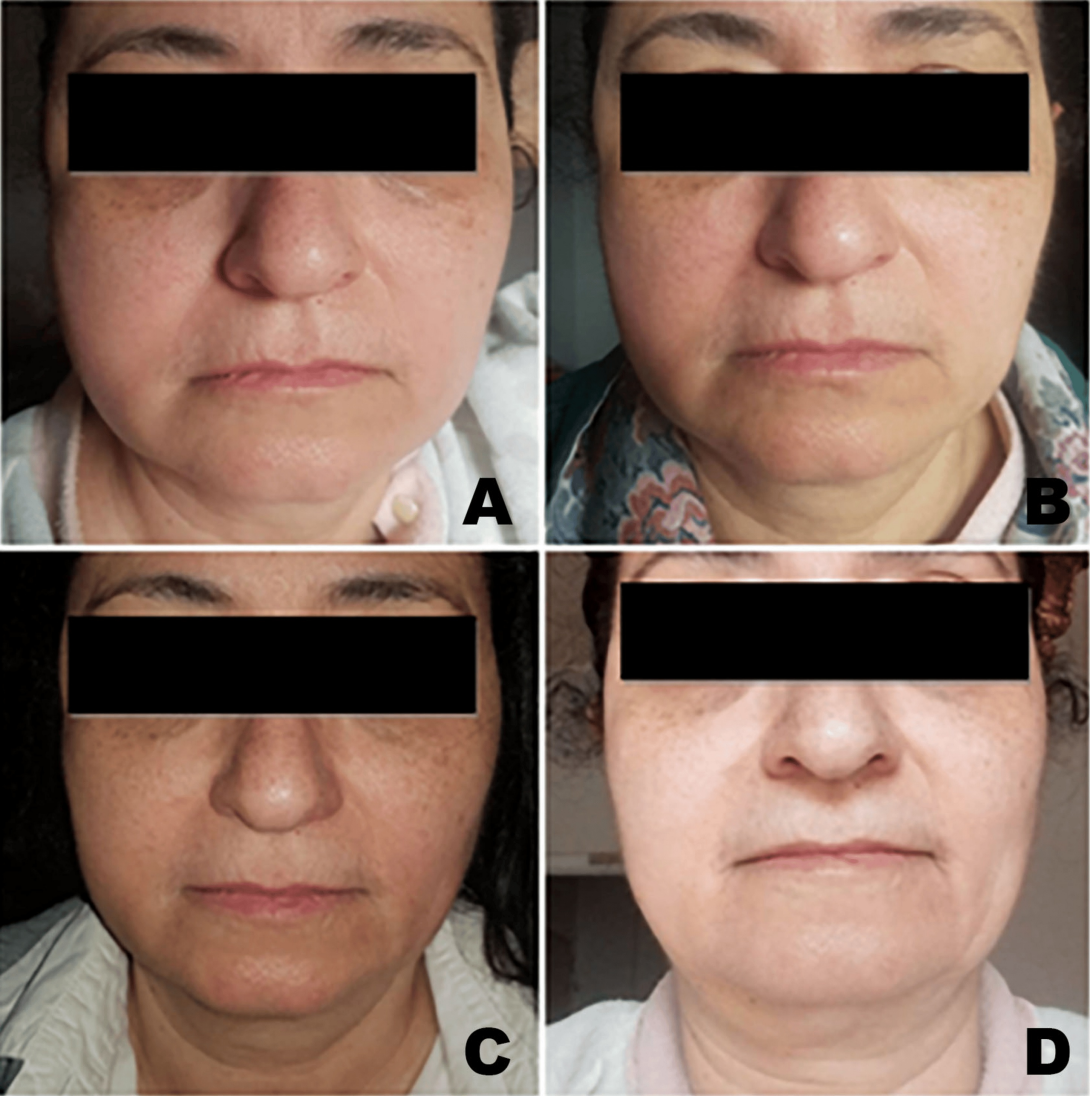
Extra-oral edema after (A) 24 hrs, (B) 48 hrs, (C) 72 hrs, (D) six days from the initial encounter

At day seven, almost total resolution of edema was observed, along with a little yellowish bruise on the lower right side of the face that appeared on the fourth day; nevertheless, there was no improvement in mouth opening, with 18 mm opening with pain on forced opening (Figure 4).

**Figure 5:**
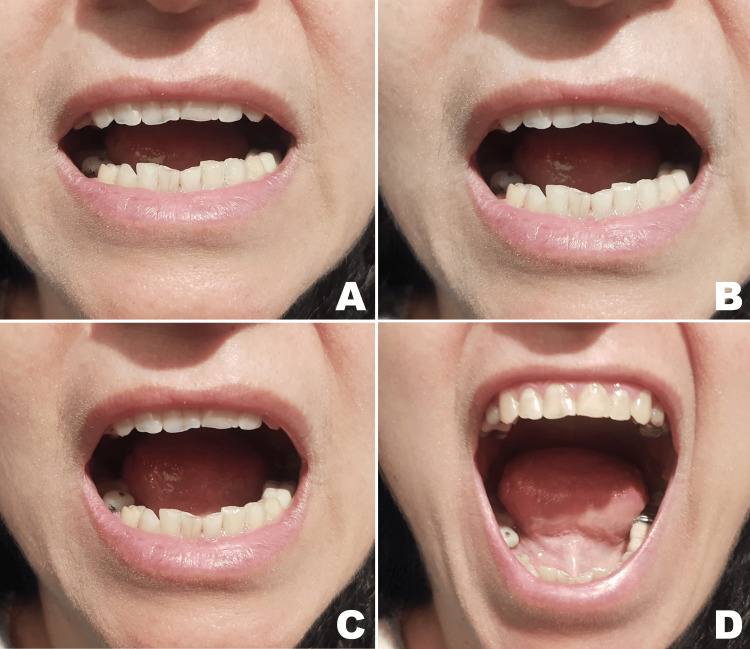
Mouth opening of (A) 13 mm at day 10, (B) 15 mm at day 12, (C) 28 mm at day 30, (D) 42 mm at day 50

Given the persistence of trismus state, new medication was introduced on day eight consisting of daily IM injections of Difen B12® (diclofenac + beclomethasone + cyancobalamin) for five days. On the same day of the first injection, total resolution of edema was noted, and the patient was a bit relieved but without improvement in mouth opening. After the second injection (day nine), the patient made extra effort during exercise to open the mouth, the pain intensified and slight edema re-emerged. As a result, the mouth opening was further reduced to 13 mm. At day 10, in addition to the third injection, Augmentin® was prescribed suspecting an infectious component; once again, edema was resolved but no improvement in mouth opening was there (13 mm). Starting from the 12th day, more emphasis was placed on the physical exercises, with increasing daily frequency. By end of the day, mouth opening reached 15 mm. The patient reported slight relief of the TMJ area upon warm application during and between exercises. She was encouraged to maintain the same protocol. By the end of the 13th day, mouth opening reached 17 mm, 19 mm by the 14th day, 28 mm by the 30th day, and, finally, 42 mm by the 50th day, marking full recovery (Figure 5). The result was maintained one month later during follow-up appointment.

## Discussion

Local anesthesia injections in dentistry are thought to be safe invasive procedures; nevertheless, complications still occur. In the present case, trismus occurred following inferior alveolar nerve block and persisted for 50 days with the relevant management.

Normal mouth opening is usually considered between 35 to 60 mm or two to three finger breadths. Many authors define trismus as occurring when a mouth opening is less than 35 mm. However, it varies from one person to another, and even by gender with men typically having a wider mouth opening [6].

Trismus can result from multiple conditions. Iatrogenic trauma (third molar extraction and intramuscular administration of anesthesia), facial or mandibular trauma, and others, all can result in acute trismus. When caused by iatrogenic factors, acute trismus usually resolves on its own, typically within a couple of weeks.

However, extended or chronic trismus is typically caused by severe trauma and delayed management. Local infections, temporomandibular disorders, surgery, radiotherapy in the head and neck, neoplastic processes, as well as connective tissue disorders (such as lupus erythematosus) can cause chronic trismus [7]. In our case, trismus lasted for several weeks and did not resolve rapidly. Delayed or inadequate management of initial trismus can allow scar tissue to develop, further restricting mandibular movement over time. As in the present case, management of prolonged trismus is more difficult than short-term trismus and requires a comprehensive approach, including aggressive physical therapy, pharmacological interventions like corticosteroids or muscle relaxants, and potentially surgical procedures to release fibrotic tissue. These findings underscore the critical importance of early recognition and prompt, effective management of trismus following IANB.

Accidental penetration of the medial pterygoid muscle can occur during the administration of this block and induce trismus associated and pain; it is worth mentioning, though, that trismus resulting from penetrating the muscle is not identical to that resulting from the intramuscular injection of anesthesia mentioned earlier (myotoxicity). Then, stretching the affected muscle results in pain, which induces an immediate reflex contraction and limitation of the mandibular opening.

When an intramuscular hematoma occurs, trismus may also arise. Hence, a hematoma in the pterygomandibular space caused by the injury to the inferior alveolar artery or vein may lead to trismus. Nevertheless, delayed trismus (after two to three days of the injection) is most likely caused by a needle-track infection [8].

Trismus is primarily treated symptomatically, with emphasis on addressing the underlying cause. Symptom-directed interventions, including heat therapy, analgesics (non-steroidal anti-inflammatory drugs (NSAIDs)) and muscle relaxants are typically administered during the acute phase. Applying moist hot towels for 15 to 20 minutes every hour is part of heat therapy. Also, a soft diet should be encouraged by the clinician for the duration of the condition [6].

Many professionals advocate the use of submucosal steroids (e.g. dexamethasone) after impacted lower third molar removal to reduce early postoperative pain and edema. Nevertheless, a systematic review and meta-analysis by O'Hare et al., suggests that, despite the fact that trismus may improve, this improvement may not be perceived as clinically significant [9].

In Nair et al., 50 patients received submucosal dexamethasone at a dose of 4 mg and 50 served as a control group for third molar surgery [10]. The submucosal dexamethasone-administered group on day two showed a statistically significant decrease in postoperative edema. On the other hand, neither group significantly outperformed the other with regard to trismus or discomfort.

In Kaewkumnert et al., submucosal dexamethasone injection (4 mg) was more effective than intramuscular injection for treating trismus following mandibular third molar extraction. Additionally, it effectively reduces postoperative pain [11].

Consequently, it has been demonstrated that the prophylactic use of dexamethasone as an anti-inflammatory drug can be an effective, safe and simple therapeutic approach for reducing edema, pain, and trismus after impacted third molars surgery.

Moreover, the oral administration of dexamethasone (20 mg) prior to and during surgical of mandibular third molars extraction may considerably reduce trismus and increase mouth opening [12].

On the other hand, it has been clarified that needle piercing does not cause trismus, according to Malamed [13]. However, if the tip of the needle unintentionally becomes barbed during injection, it may tear the medial pterygoid muscle fibers during needle withdrawal, causing muscular spasm and, eventually, trismus [13].

Additionally, according to White et al., the act of surgery initiates a series of events that include the release of inflammatory mediators, causing a temporary constriction of arterioles followed by vasodilation, increased blood flow, an increase in the permeability of post-capillary venules, and fluid leakage into the surrounding tissue [14]. Consequently, trismus is a condition that results from the contraction of these muscle fibers after an inflammatory process [15].

In contrast, our case found a decrease in mouth opening on the initial postoperative days and edema on the right side, followed by an increase in pain threshold after an IANB administration. Subsequently, the mouth opening exhibited a progressive increase by postoperative day 13 and by full recovery by day 50, as well as a decrease in pain and edema after the administration of our protocols.

A major limitation in our case is represented by the fact that there was early gradual increase in mouth opening by postoperative day 13, yet full recovery was attained by day 50, suggesting that the trismus may have been due to soft tissue trauma rather than a significant structural injury or a chronic issue. However, the long recovery period might obscure whether the treatment protocol or the natural healing process was primarily responsible for recovery, especially in the absence of existing literature on definitive intervention. Therefore, future case series or studies with comparative treatment groups would provide more robust conclusions about the effectiveness of the intervention.

Thus, multifaceted approach with TMJ exercises and the administration of antibiotics, anti-inflammatory drugs, analgesics, and muscle relaxants can effectively reduce trismus and improve the overall outcome of the process.

## Conclusions

Iatrogenic trismus presents an overlooked condition in everyday practice. Despite being usually temporary, it may negatively affect a patient’s quality of life and cause considerable derangement. Management of trismus remains unstandardized with limited definitive treatment available. Therefore, recognition of primary culprit is essential, whether the injection, surgery, infection or others. Then, a step-up approach may be best suitable for such cases, depending on the cause, starting with least reassurance and supportive treatment, reaching long medication courses and extensive physical therapy. Eventually, future research should investigate trismus epidemiology, exact causes and best evidence-based management modalities in treatment as well as prevention of trismus entirely.
